# The Putative bZIP Transcripton Factor BzpN Slows Proliferation and Functions in the Regulation of Cell Density by Autocrine Signals in *Dictyostelium*

**DOI:** 10.1371/journal.pone.0021765

**Published:** 2011-07-07

**Authors:** Jonathan E. Phillips, Eryong Huang, Gad Shaulsky, Richard H. Gomer

**Affiliations:** 1 Department of Biochemistry and Cell Biology, Rice University, Houston, Texas, United States of America; 2 Department of Molecular and Human Genetics, Baylor College of Medicine, Houston, Texas, United States of America; 3 Department of Biology, Texas A&M University, College Station, Texas, United States of America; Cardiff University, United Kingdom

## Abstract

The secreted proteins AprA and CfaD function as autocrine signals that inhibit cell proliferation in *Dictyostelium discoideum*, thereby regulating cell numbers by a negative feedback mechanism. We report here that the putative basic leucine zipper transcription factor BzpN plays a role in the inhibition of proliferation by AprA and CfaD. Cells lacking BzpN proliferate more rapidly than wild-type cells but do not reach a higher stationary density. Recombinant AprA inhibits wild-type cell proliferation but does not inhibit the proliferation of cells lacking BzpN. Recombinant CfaD also inhibits wild-type cell proliferation, but promotes the proliferation of cells lacking BzpN. Overexpression of BzpN results in a reduced cell density at stationary phase, and this phenotype requires AprA, CfaD, and the kinase QkgA. Conditioned media from high-density cells stops the proliferation of wild-type but not *bzpN^−^* cells and induces a nuclear localization of a BzpN-GFP fusion protein, though this localization does not require AprA or CfaD. Together, the data suggest that BzpN is necessary for some but not all of the effects of AprA and CfaD, and that BzpN may function downstream of AprA and CfaD in a signal transduction pathway that inhibits proliferation.

## Introduction

During organismal development, tissues of characteristic shapes and sizes are generated from progenitor cell populations through proliferation and differentiation. Although great insights have been gained regarding the mechanisms that pattern tissues during development, much remains to be understood about how the sizes of tissues are established and maintained [1], [2]. Proliferation-inhibiting signals that are produced by and act on the same population of cells can regulate the size of tissues by effectively halting proliferation once a certain cell number has been reached [3]. For instance, the TGF-ß superfamily member myostatin is secreted by skeletal muscle and inhibits the proliferation of muscle cell progenitors, functioning to limit muscle size [4]. Although there is evidence of such signals operating in many cell types [5], [6], [7], few signaling molecules of this sort have been identified and little is known about intracellular signaling pathways that couple such signals to the inhibition of proliferation. Some tumors appear to secrete factors that inhibit the proliferation of tumor cells [8], and such factors secreted by primary tumors may inhibit the proliferation of metastases [9], [10]. Thus, identification and characterization of autocrine signals that inhibit proliferation may be useful in developing therapies to limit tumor growth and to keep metastases in a dormant state.

The social amoeba *Dictyostelium discoideum* is an excellent model system for the study of extracellular signals that inform individual cells about local cell numbers [11], [12], [13]. When nutrients are abundant, *Dictyostelium* exists as unicellular amoebae. However, under conditions of starvation, and if the density of starving cells is above a certain threshold as indicated to the cells by high levels of CMF, a protein secreted by starving cells [12], cells secrete and respond to the chemoattractant cAMP [14], leading to the formation of streams of cells that aggregate to form groups of about 20,000 cells [15]. These aggregates develop into multicellular fruiting body structures composed of differentiated cell types and consisting of an approximately 2 millimeter high stalk supporting a ball of spores [16]. The stalk structure aids in dispersal of spores to areas where nutrients are present [17], resulting in spore germination and resumption of vegetative growth.

During vegetative growth, *Dictyostelium* cells secrete the proteins AprA and CfaD, which inhibit the proliferation of *Dictyostelium* cells in a concentration-dependent manner [18], [19]. Extracellular levels of AprA and CfaD increase as a function of cell density, and cells lacking either AprA or CfaD proliferate more rapidly than wild-type cells, are multinucleate, and reach a higher stationary density than wild type [18], [19]. The addition of either recombinant AprA (rAprA) or rCfaD to wild-type cells slows proliferation, though cells lacking AprA are not slowed by rCfaD [19], and cells lacking CfaD are not slowed by rAprA [20], suggesting that these proteins require each other for activity. Cells lacking AprA or CfaD accumulate mass on a per nucleus basis at a rate like wild-type cells, indicating that AprA and CfaD regulate proliferation but not cell growth [18], [19]. As cells tend to starve when they reach high cell densities, slowed proliferation due to AprA and CfaD combined with unchanged cell growth may provide cells with stored resources that aid in survival under conditions of starvation. This is supported by the observations that *aprA^−^* and *cfaD^−^* cells die more rapidly than wild type after reaching stationary density, and that *aprA^−^* and *cfaD^−^* cells produce fewer viable spores than wild type following starvation-induced development [18], [19].

Little is known about the signal transduction pathway downstream of AprA and CfaD. The G protein complex subunits Ga8, Ga9, and Gß are necessary for the activity of AprA [21], suggesting that AprA may signal through a G protein-coupled receptor. Additionally, the ROCO kinase QkgA is necessary for the inhibition of proliferation by AprA and CfaD, and *qkgA^−^* cells phenocopy the rapid proliferation, high stationary density, and multinuclearity of *aprA^−^* and *cfaD^−^* cells [22]. However, *qkgA^−^* cells do not show defects in spore viability [22], indicating that AprA and CfaD affect spore viability by a mechanism independent of QkgA.

Basic leucine zipper (bZIP) transcription factors are a large family of proteins that function in a wide range of signal transduction pathways [23], [24] and are defined by an approximately 30 amino-acid sequence with a leucine residue at every seventh position, which mediates homo- or heterodimerization through alpha-helical interactions [25] and a stretch of adjacent basic residues that mediate DNA binding [26]. The *Dictyostelium* genome encodes 19 predicted bZIP transcription factors. Little is known regarding the function of the majority of these bZIP proteins, though the bZIP proteins DimA and DimB are downstream effectors of the prestalk cell fate-specifying signal DIF and translocate to the nucleus in response to this signal [27], [28], [29]. Here we report that the putative bZIP transcription factor BzpN plays a role in AprA- and CfaD-mediated inhibition of proliferation. *bzpN^−^* cells proliferate rapidly, show aberrant responses to rAprA and rCfaD, and are multinucleate. However, *bzpN^−^* cells do not show defects in spore viability and do not proliferate to a higher stationary density than wild type. Thus BzpN mediates a subset of the effects of AprA and CfaD.

## Materials and Methods

### Deletion mutation of *bzpN* and BzpN-GFP construct

Regions upstream and downstream of the *bzpN* gene, which flank the entire open reading frame of the gene, were PCR-amplified (primers for upstream amplification: 5′-TCCCGCGGTTTTTGGATTACGGCACACA and 5′-TCAAGCTTCCAGGTGATGAAGGGATTGA; primers for downstream amplification: 5′-TCGGTACCAACCGCAGCCTCTACTTCAA and 5′-TCCCGCGGCAACCGATTTTACCCTCACAA). Both PCR fragments were purified and digested with the restriction endonuclease SacII, and then the two fragments were ligated together and PCR was done using the primers 5′-TCGGTACCAACCGCAGCCTCTACTTCAA and 5′-TCAAGCTTCCAGGTGATGAAGGGATTGA to amplify the ligated product. This PCR product was digested and cloned into the KpnI-HindIII sites of the pLPBLP-ΔSacII plasmid [30] to generate the null knockout vector. For transformation of AX4 cells, 10 µg of the knockout vector was linearized using SacII and then electroporated into AX4 cells [31]. Cells were selected in HL5 medium supplemented with 10 µg/ml Blasticidin S for at least 10 days. Colonies were plated along with bacteria (*Klebsiella aerogenes*) on SM agar plates for plaque formation. Colony PCR was performed on the resulting plaques to identify knockout mutants. Colonies that yielded a PCR product using the diagnostic primer (5′-GGCAAAAAGTTGACAACAACAA) and a BSR cassette internal primer (5′-TCTTGTTGAGAAATGTTAAATTGATACCA) were chosen. The successful gene disruption was further verified by RT-PCR with the primers ATGGTAGCGCTGATCCAAGT and GCGAGATTTCACCATCCAAT on RNA samples collected from both wild-type and *bzpN^−^* cells during vegetative growth. Total RNA samples were treated with DNase I to eliminate traces of genomic DNA, and then reverse-transcribed into cDNA with an oligo-dT primer (Invitrogen). Real-time PCR was performed using a Biorad (MJ research) Opticon3 system as described [27].

To generate a *bzpN-GFP* transgene, the *bzpN* open reading frame lacking the stop codon was amplified by PCR from wild-type vegetative cDNA with the primers 5′-GGACTAGTATGTATCAAAGTATTCCTCAACAAGG and 5′-GGACTAGTAGTATAAGTTGGATCAACATAAGAATAAAG, which incorporate terminal SpeI sites. This PCR product was cloned into the pGEM-T vector (Promega). The *bzpN* open reading frame was then isolated by SpeI digestion and gel purification, and ligated into the SpeI site of the *Dictyostelium* extrachromosomal expression vector pDM323 [32] to generate an open reading frame encoding BzpN with GFP fused to the C-terminus. Correct orientation of the *bzpN* open reading frame within the vector was confirmed by restriction mapping. This vector was transformed into *Dictyostelium* cells following Manstein et al [33].

### Cell culture, proliferation curves, inhibition assays, immunoblotting, mass and protein measurement, colony size measurement, and spore viability

Cell culture, proliferation curves in shaking culture, proliferation on lawns of bacteria, and nuclei counts were done according to [18]. Comparison of extracellular AprA and CfaD by immunoblotting, measurement of mass and protein content, colony size measurement on bacterial lawns, and spore viability assays were done following [22]. Silver staining of conditioned media was done following [13]. rAprA and rCfaD inhibition assays were done following [22], except that cells were incubated with rAprA or rCfaD for 48 hours before measurement of cell density.

To measure proliferation in response to stationary phase conditioned media, wild-type cells were grown to a density of 20–25×10^6^ cells/mL in HL5. Cells were removed by centrifugation at 800× g for 3 minutes at room temperature and the conditioned medium supernatant was collected and sterilized by passage through a 0.2 micron filter (Pall, Ann Arbor, MI). Wild-type, *bzpN^−^*, or *bzpN^−^/bzpN-GFP* cells were then resuspended in this conditioned media in 5 mL volumes at 0.5×10^6^ cells/mL, and cell density was determined by hemocytometer after a 24 hour incubation in shaking culture at 22°C.

### Fluorescence microscopy

To determine the effect of conditioned media on BzpN-GFP localization, conditioned media from wild-type, *aprA^−^*, and *cfaD^−^* cells at a density of 20–25×10^6^ cells/mL was collected as described above. *bzpN^−^/act15::bzpN-GFP* cells at a density of 0.5–4×10^6^ cells/mL were resuspended in 0.5 ml volumes at 0.5×10^6^ cells/mL in the media in which they had been growing, in fresh HL5, or in conditioned media from wild-type, *aprA^−^*, or *cfaD^−^* cells. These cultures were incubated overnight at 22°C in shaking culture, and then 200 µL of each culture was allowed to settle in one well of an 8-well chamber slide (Nunc) for 30 minutes. The medium was removed and cells were then fixed in 4% paraformaldehyde in PBS pH 7.4 for 15 minutes, permeablized with 0.1% NP-40 in PBS pH 7.4 for 2 minutes, and washed three times in PBS pH 7.4. Cells were then mounted in Vectashield mounting media with DAPI (Vector, Burlingame, CA) and imaged with an Olympus FV1000 fluorescence microscope. To examine BzpN-GFP localization in comparison to lysosomal or mitochondrial localization, *bzpN^−^/bzpN-GFP* cells at 0.5–4×10^6^ cells/mL in shaking culture were resuspended in low-fluorescence media [34] at 0.5×10^6^ cells/mL with either 500 nM Lysotracker Red dye (Invitrogen) or 500 nM Mitotracker Red dye (Invitrogen) and incubated in shaking culture at 22°C for 2 hours. Cells were then washed once in low-fluorescence media and then fixed and imaged as described above.

## Results

### BzpN regulates proliferation

**Figure 1:**
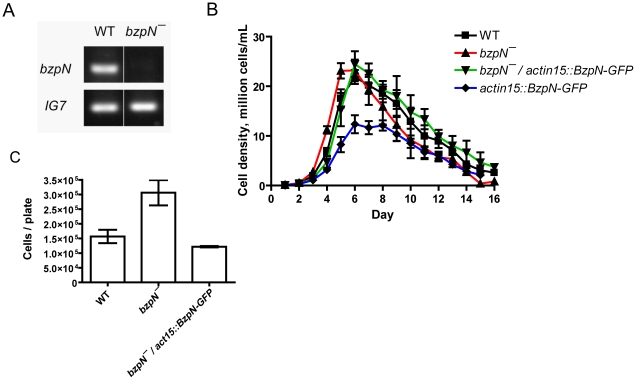
BzpN slows the proliferation of cells. (A) The *bzpN* transcript is not detected in *bzpN^−^* cells during vegetative growth as assayed by RT-PCR. The mitochondrial ribosomal RNA IG7 serves as a control. (B) Log phase cells were diluted to 1×10^5^ cells/mL and grown in shaking culture. Values are means ± SEM (n≥4) for all conditions. WT indicates wild type. All strains are in the AX4 background. (C) 1000 cells were spread on agar plates with bacteria. After 24 hours, the *Dictyostelium* cells were collected and counted. Values are means ± SEM (n≥3). The differences in cell numbers between wild-type and *bzpN^−^* and between *bzpN^−^* and *bzpN^−^/bzpN-GFP* are significant (p<0.05, one-way ANOVA, Tukey's test).

To gain insight into the function of basic leucine zipper transcription factors encoded in the *Dictyostelium* genome, genes encoding bZIP proteins were targeted for disruption by homologous recombination [27]. The *BzpN* gene encodes a 999-amino acid protein that has 33% identity and 56% similarity over a 59-amino acid segment to the human bZIP protein Nrf2, which mediates cellular responses to oxidative stress [35]. A *BzpN* null mutant was generated and confirmed by diagnostic PCR and RT-PCR (Figure 1A). *bzpN^−^* cells exhibited rapid proliferation as compared to wild-type cells. To determine if the rapid proliferation was due to loss of BzpN, we constructed an extrachromosomal vector in which the constitutive *actin15* promoter drives expression of BzpN with GFP fused to the C-terminus, transformed wild-type and *bzpN^−^* cells with this vector, and examined the proliferation of these cell lines. In shaking culture, *bzpN^−^* cells showed faster proliferation than wild-type cells during the logarithmic phase of proliferation and had a significantly faster doubling time (Figure 1B and Table 1). However, *bzpN^−^* cells did not proliferate to a higher cell density than wild type (Table 1), in contrast with *aprA^−^* and *cfaD^−^* cells [18], [19]. The expression of BzpN-GFP in *bzpN^−^* cells resulted in a proliferative phenotype and doubling time similar to wild-type cells, strongly suggesting that the rapid proliferation phenotype of *bzpN^−^* cells is due specifically to the absence of BzpN. Although *bzpN^−^/act15::bzpN-GFP* cells did not show a reduced maximum cell density as compared to wild type, expression of BzpN-GFP in wild-type cells resulted in a significantly reduced maximum cell density (Table 1, Figure 1B), suggesting that dosage of BzpN affects proliferation. *bzpN^−^* cells also proliferated faster than wild-type cells when grown on bacterial lawns, and this rapid proliferation was rescued by the expression of BzpN-GFP (Figure 1C). Together, these results indicate that BzpN functions to slow the proliferation of cells during exponential growth.

**Table 1:** The effect of BzpN on the doubling time and stationary density of cells.

Genotype	Doubling time, hours	Maximum observed cell density, 10^6^ cells/mL
Wild type	12.1±0.4	22.6±1.8
*bzpN^−^*	10.5±0.2*	25.2±1.3
*bzpN^−^/actin15::bzpN-GFP*	13.7±0.9	25.0±2.5
*actin15::bzpN-GFP*	14.0±0.4	14.4±0.6*

Doubling times and stationary densities were calculated for the proliferation curves in Figure 1. Values are mean ± SEM from four or more independent experiments.

*indicates that the difference between the value and the wild-type value is significant with p<0.05 (one-way ANOVA, Tukey's test).

### *bzpN^−^* cells do not show a deficiency in AprA or CfaD accumulation

**Figure 2:**
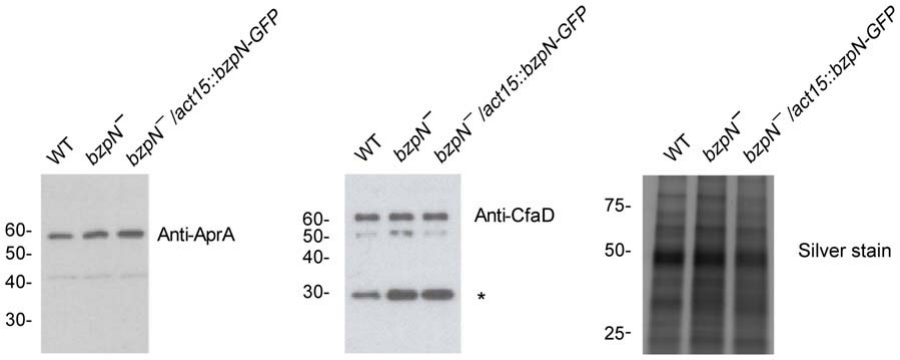
*bzpN^−^* cells secrete AprA and CfaD. Cells were grown to 12±1×10^6^ cells/mL in shaking culture and conditioned medium was collected and assayed by Western blot with anti-AprA or anti-CfaD antibodies. Asterisk indicates a 27 kDa breakdown product of CfaD. The intensity of this 27 kDa band and the light band at 50 kDa were variable between experiment replicates. Silver staining of conditioned media samples was done as a loading control. Numbers at the left indicate molecular mass in kDa. Data are representative of three independent experiments.

One explanation for the rapid proliferation of *bzpN^−^* cells could be reduced extracellular AprA and/or CfaD accumulation due to aberrant expression, secretion, or degradation of these proteins. However, wild-type and *bzpN^−^* cells showed similar levels of extracellular AprA and CfaD at both high and low cell densities (Figure 2 and data not shown). A 27 kD breakdown product of CfaD [19] appeared at higher levels in both *bzpN^−^* and *bzpN^−^/act15::bzpN-GFP* conditioned media as compared to wild-type, although this difference likely does not significantly affect proliferation, as suggested by the similar proliferation rates of wild-type and *bzpN^−^/act15::bzpN-GFP* cells. Silver staining of conditioned media samples showed similar protein levels, indicating equal loading and showing wild-type levels of extracellular protein accumulation in *bzpN^−^ cells*. These results suggest that BzpN is not essential for normal extracellular accumulation of AprA and CfaD, and that the fast proliferation of *bzpN^−^* cells is not due to a lack of extracellular AprA or CfaD.

### *bzpN^−^* cells show aberrant responses to AprA and CfaD

**Figure 3:**
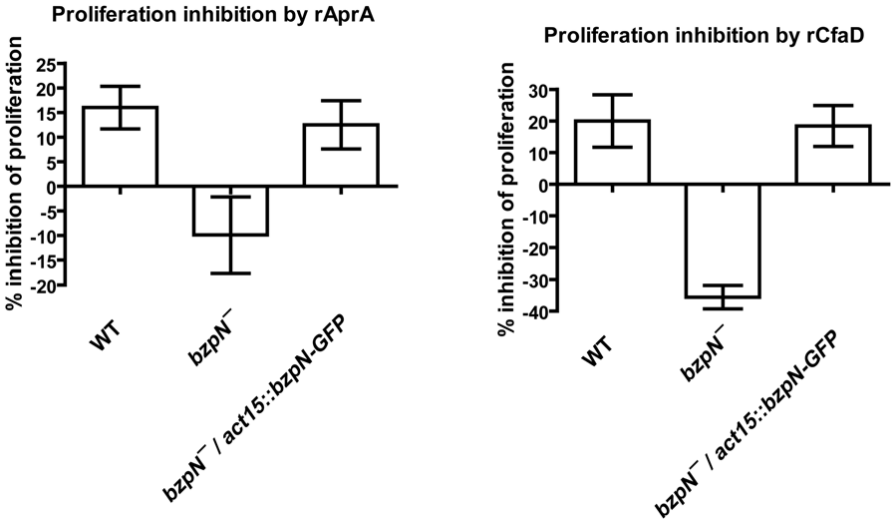
*bzpN^−^* cells show aberrant responses to AprA and CfaD. Cells were incubated with either rAprA, rCfaD, or an equivalent volume of buffer. Cell densities were measured after 48 hours, and the percent inhibition of proliferation by AprA or CfaD as measured against a buffer control was calculated. Values are mean ± SEM (n≥4). For both AprA and CfaD, differences between wild type and *bzpN^−^* cells are significant (p<0.05, 1-way ANOVA, Tukey's test), whereas differences between wild type and rescue are not. For *bzpN^−^* cells, rCfaD but not rAprA significantly increased proliferation (p<0.05, paired t-test).

If BzpN is a component of an AprA/CfaD signal transduction pathway, *bzpN^−^* cells could be insensitive to the proliferation-inhibiting effects of AprA or CfaD. To test this possibility, we incubated wild-type, *bzpN^−^*, or *bzpN^−^/act15::bzpN-GFP* cells at low density with rAprA or rCfaD and determined the ability of these proteins to inhibit proliferation. As previously observed, rAprA and rCfaD inhibited the proliferation of wild-type cells [21], [22] (Figure 3). However, rAprA had no significant effect on the proliferation of *bzpN^−^* cells, whereas rCfaD significantly increased *bzpN^−^* cell proliferation. In contrast, both rAprA and rCfaD inhibited the proliferation of *bzpN^−^/act15::bzpN-GFP* cells similar to wild-type. The increased proliferation of *bzpN^−^* cells in response to rCfaD may indicate the existence of a proliferation-promoting branch of the CfaD signal transduction pathway that serves to attenuate the proliferation-inhibiting effects of CfaD and that this proliferation-promoting pathway remains active in *bzpN^−^* cells. These results indicate that BzpN is necessary for the proliferation-inhibiting activity of AprA and CfaD and suggest that BzpN may function downstream of AprA and CfaD in a signal transduction pathway.

### *bzpN^−^* cells are multinucleate and accumulate mass at a rate like wild type

Both *aprA^−^* and *cfaD^−^* cells have more nuclei per cell than wild type, which may be caused by rapid mitosis without a concomitant increase in the rate of cytokinesis [18], [19]. To determine whether *bzpN^−^* cells share this phenotype, we examined the number of nuclei per cell for wild type, *bzpN^−^* cells, and *bzpN^−^/act15::bzpN-GFP* cells. *bzpN^−^* cells had significantly more nuclei per cell than wild type, whereas *bzpN^−^/act15::bzpN-GFP* cells showed no significant difference in nuclei per cell compared to wild type (Table 2). These results indicate that, like AprA and CfaD, BzpN reduces the average number of nuclei per cell.

**Table 2:** The effect of BzpN on the mass, protein, and nuclei content of cells.

	Per 10^7^ cells		% cells with *n* nuclei			Nuclei/100 cells	Per 10^7^ nuclei	
Genotype	Mass (mg)	Protein (mg)	1	2	3+		Mass (mg)	Protein (mg)
Wild type	9.0±0.3	0.51±0.02	44±9	45±7	10±5	168±9	5.4±0.2	0.30±0.01
*bzpN^−^*	10.7±0.1*	0.63±0.02*	27±4*	54±7	19±9	199±10*	5.4±0.2	0.31±0.02
*bzpN^−^/actin15::bzpN-GFP*	9.0±0.2	0.49±0.01	50±5	38±2	12±3	168±6	5.4±0.2	0.29±0.01

Mass and protein content were determined as described in the Materials and Methods. Values are the mean ± SEM from three or more independent experiments.

*indicates that the difference between the value and the wild-type value is significant with p<0.05 (one-way ANOVA, Tukey's test).

Although growth (the accumulation of mass) is essential for continued proliferation, cell growth and proliferation can be regulated independently [36]. *aprA^−^* and *cfaD^−^* cells show an accumulation of mass per nucleus like that of wild type, suggesting that AprA and CfaD regulate proliferation but not growth [18], [19]. To determine the effect of BzpN on growth, we measured the mass and protein content of log phase cells and calculated the accumulation of mass and protein per cell and per nucleus using the measured doubling times (Table 1) and nuclei counts (Table 2). AX4, the parental strain of *bzpN^−^*, was more massive and had more protein on a per cell basis than we have previously observed for AX2, the parental strain of our previously characterized mutants [22]. AX4 also had more nuclei per cell than AX2 [18], though mass and protein levels on a per nucleus basis for AX4 are similar to what we have observed for AX2 [22]. *bzpN^−^* cells were significantly more massive and had a higher protein content than wild-type or *bzpN^−^/act15::bzpN-GFP* cells (Table 2), though these differences were lost when mass and protein content were considered on a per nucleus basis. We then examined mass and protein accumulation as a function of time by dividing the observed mass and protein measurements by the measured doubling times, yielding the accumulation of mass or protein per hour. Mass and protein accumulation per cell was greater in AX4 than we have seen in AX2, though mass and protein accumulation per nucleus for AX4 are similar to what we have seen for AX2 [22]. *bzpN^−^* cells accumulated mass, protein, and nuclei more rapidly than wild-type or *bzpN^−^/act15::bzpN-GFP* cells (Table 3). However, differences in the accumulation of mass or protein on a per nucleus basis were not significant between wild-type and *bzpN^−^* cells (Table 3). These results suggest that BzpN, like AprA and CfaD, regulates proliferation but does not significantly regulate growth on a per nucleus basis.

**Table 3:** The effect of BzpN on the mass and protein accumulation of cells.

	Per 10^7^ cells per hour			Per 10^7^ nuclei per hour	
Genotype	Mass (mg)	Protein (µg)	Nuclei, ×10^−5^	Mass (mg)	Protein (µg)
Wild type	0.74±0.03	42±2	14±1	0.44±0.02	25±2
*bzpN^−^*	1.02±0.02*	59±2*	19±1*	0.51±0.02	30±2
*bzpN^−^/actin15::bzpN-GFP*	0.66±0.04	36±2	12±1	0.39±0.03	21±2

Mass and protein values from Table 2 were divided by the observed doubling time of the respective genotype. Doubling times were calculated as described in Materials and Methods. Values are the mean ± SEM from three or more independent experiments.

*indicates that the difference between the value and the wild-type value is significant with p<0.05 (one-way ANOVA, Tukey's test).

### AprA, CfaD, and QkgA are necessary for proliferation inhibition by BzpN-GFP overexpression

**Figure 4:**
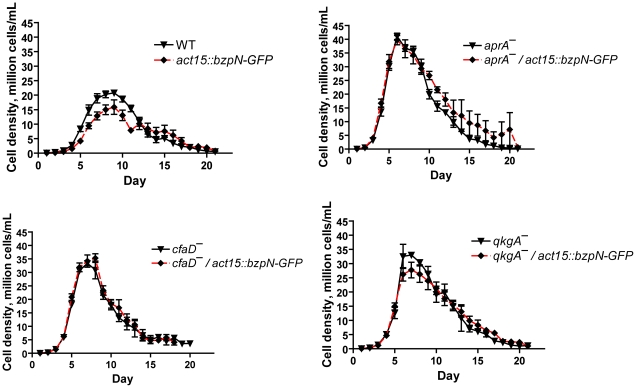
AprA, CfaD, and QkgA are required for inhibition of proliferation by BzpN. Proliferation curves were done as described in Figure 1. Values are mean ± SEM, n≥3. Expression of BzpN-GFP in wild-type cells resulted in a significant decrease in cell density on days 5 through 8 with p<0.05, whereas expression of BzpN-GFP in *aprA^−^, cfaD^−^, or qkgA^−^* cells did not cause a significant reduction in cell density at any measured timepoint (t-test). WT indicates wild type. All strains are in the AX2 background. Data for strains expressing BzpN-GFP are the average of two independent transformant clones.

Overexpression of BzpN in the wild-type background results in slow proliferation and a low stationary phase density (Figure 1B). To test whether AprA, CfaD, or QkgA are necessary for proliferation inhibition by BzpN, we expressed BzpN-GFP in cells lacking these proteins, confirmed expression by fluorescence microscopy, and examined the proliferation of these strains in parallel with the parental strains. As our *aprA^−^*, *cfaD^−^*, and *qkgA^−^* cells are in the AX2 genetic background, we expressed BzpN-GFP in AX2 cells as a control, and saw a similar reduction in maximum cell density as in Figure 1B, where the AX4 strain was used (Figure 4). Whereas expression of BzpN-GFP in wild-type cells resulted in significantly lower cell densities during proliferation as compared to untransformed wild-type cells, expression of BzpN-GFP in *aprA^−^*, *cfaD^−^*, or *qkgA^−^* cells resulted in no significant decrease in cell density during proliferation as compared to untransformed parental cells (Figure 4). These results suggest that AprA, CfaD, and QkgA are essential for the proliferation-inhibiting activity of BzpN.

### BzpN does not affect the production of spores or the ability of cell colonies to expand

*aprA^−^* and *cfaD^−^* cells yield fewer viable, detergent-resistant spores than wild type following multicellular development, indicating that AprA and CfaD promote the development of viable spores [18], [19]. We tested the effect of BzpN on spore development by allowing wild-type, *bzpN^−^*, and *bzpN^−^/act15::bzpN-GFP* cells to develop, collecting and counting spores, and plating detergent-treated spores on lawns of bacteria. For all genotypes, the number of spores collected was higher than the number of cells allowed to develop (Table 4). This is a higher spore yield than we have previously observed, and may be due to the high percentage of multinuclear cells in the Ax4 background, or a round of cell proliferation during development, as has been previously observed [37]. Differences between any two genotypes for either the number of spores collected or the number of viable detergent-treated spores were not significant (Table 4). These results suggest that BzpN does not significantly affect spore viability and that AprA and CfaD affect spore development by a mechanism independent of BzpN.

**Table 4:** The effect of BzpN on spore viability.

Genotype	Visible spores after development as a percent of input cell number	Detergent-resistant spores as a percent of total spores
Wild type	133±32	54±6
*bzpN^−^*	183±8	74±5
*bzpN^−^/act15::BzpN-GFP*	163±31	74±8

10^7^ cells were allowed to develop on filter pads and spores were collected in buffer by repeated washing of the filter pad using a pipette. The density of visible spores in buffer was determined by hemocytometer and the total number of collected spores was calculated. Spores were then treated with detergent, and serial dilutions of detergent-treated spores were plated on SM/5 plates in association with bacteria. The number of resultant plaques in the bacterial lawn was used to calculate the total number of detergent resistant spores as a percent of input cell number. Values are the mean ± SEM from three independent experiments.

Despite the fact that *aprA^−^* and *cfaD^−^* cells proliferate rapidly, colonies of these cells on a lawn of bacteria expand less rapidly than wild-type colonies [22], indicating that AprA and CfaD may function to facilitate the dispersal of groups of cells. To determine whether *bzpN^−^* cells expand slowly as colonies on a lawn of bacteria, we plated serial dilutions of wild-type and *bzpN^−^* cells mixed with *K. aerogenes* bacteria on SM/5 plates and measured the diameter of well-spaced plaques daily. No significant differences were observed between the sizes of wild-type and *bzpN^−^* plaques (data not shown), suggesting that AprA and CfaD facilitate the expansion of colonies in a manner independent of BzpN.

### Conditioned media from wild type, *aprA^−^*, or *cfaD^−^* cells induce nuclear localization of BzpN-GFP

**Figure 5:**
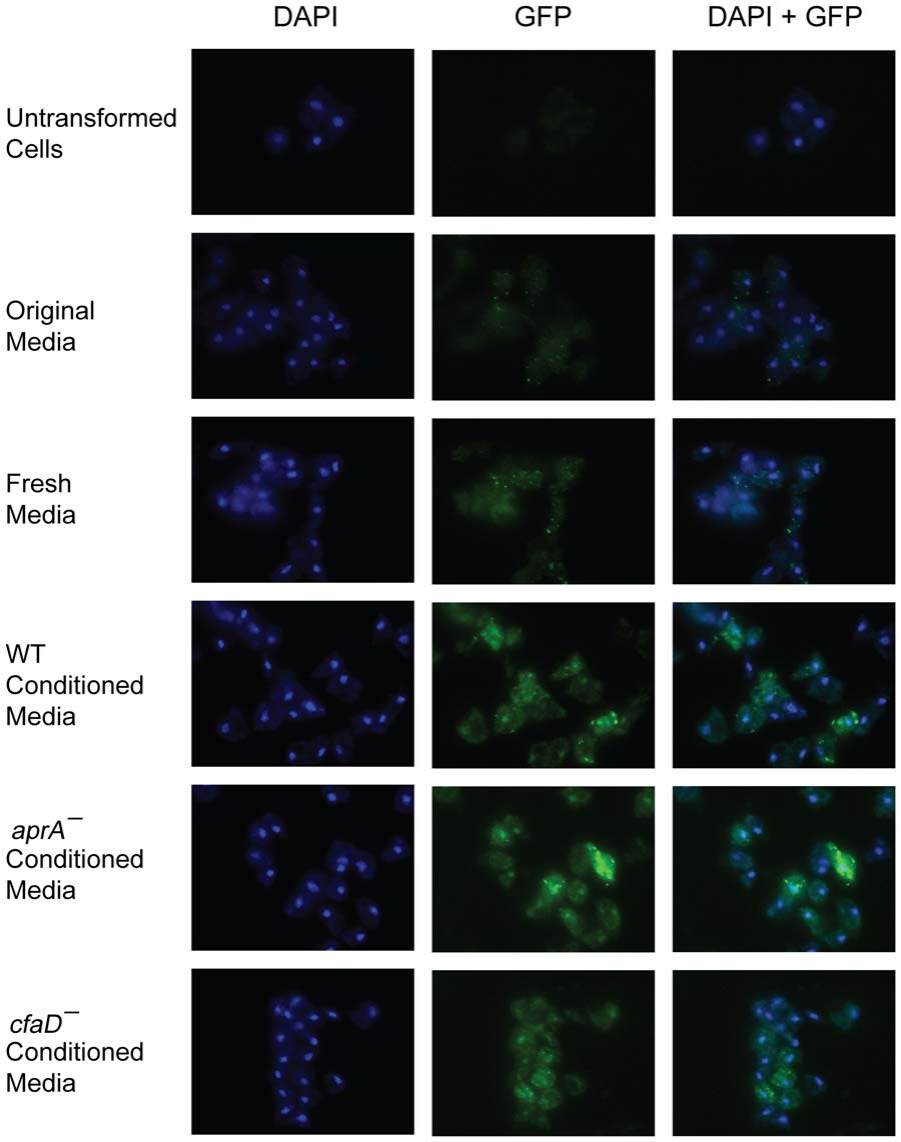
BzpN-GFP fusion proteins show a punctate localization at low density and a nuclear localization in response to conditioned media from high-density cells. BzpN-GFP expressing cells at low density were collected by centrifugation and resuspended in either the original media in which they had been growing, fresh media, or conditioned media from cells of the indicated genotype. Cells were incubated in shaking culture overnight, and then a sample of the culture was allowed to settle on glass chamber slides for 30 minutes. The cells were subsequently fixed, stained with DAPI, and imaged. Scale bar: 20 µm.

Basic leucine zipper transcription factors are known to translocate to the nucleus in response to extracellular signals in *Dictyostelium* [27]. To test whether AprA, CfaD, or conditioned media from high density cells are sufficient for nuclear translocation of BzpN, we added rAprA, rCfaD, both proteins, or conditioned medium from high density cells to low density cells expressing BzpN-GFP and examined localization of the BzpN-GFP fusion protein. In low-density cells, the fusion protein showed a punctate localization (Figure 5) that did not co-localize with either mitochondria or lysosomes (data not shown). The addition of AprA, CfaD, or both proteins resulted in no change in BzpN-GFP localization (data not shown). However, the addition of conditioned medium from high-density wild-type, *aprA^−^*, or *cfaD^−^* cells resulted in a nuclear localization of BzpN-GFP as judged by colocalization with DAPI staining (Figure 5). These results suggest that a factor present in conditioned media from high-density cells that is not AprA or CfaD mediates localization of BzpN to the nucleus.

### BzpN prevents proliferation of cells in the presence of conditioned media from high-density cell cultures

**Figure 6:**
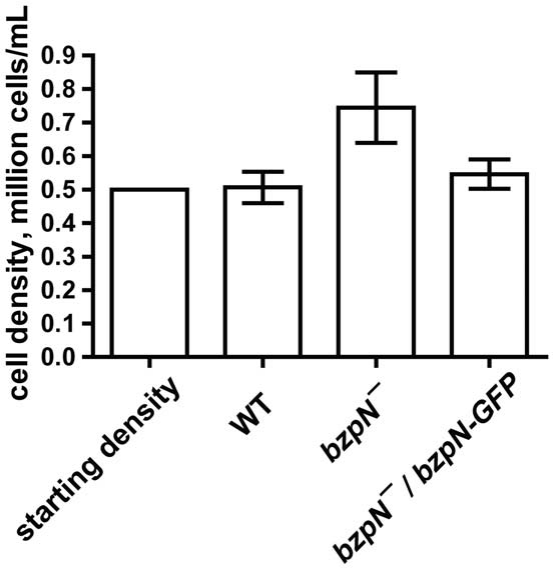
*bzpN^−^* cells but not wild-type cells proliferate in the presence of conditioned medium from cells at high density. Log phase cells were collected by centrifugation and resuspended at 0.5×10^6^ cells/mL in conditioned media from wild-type cells grown to a density of 20×10^6^ cells/mL. Cell densities were measured after 24 hours in shaking culture. Values are mean ± SEM (n = 5). The differences in cell densities between *bzpN^−^* and either wild-type or *bzpN^−^/act15::bzpN-GFP* cells are significant (p<0.05, repeated measures ANOVA, Tukey's test).

Conditioned media from cells at stationary density prevents proliferation, and this inhibition may depend on an unidentified small molecule present in conditioned media [38]. As BzpN negatively regulates proliferation and BzpN-GFP localizes at the nucleus in response to conditioned media from high-density cells, we tested whether BzpN was necessary for this inhibition of proliferation. When conditioned media from stationary cells was added to wild-type cells at low cell density, we saw no increase in cell density after 24 hours (Figure 6). However, under the same conditions, *bzpN^−^* cells showed a statistically significant increase in cell density, and this increase was not observed in *bzpN^−^/act15::bzpN-GFP* cells. Cells resuspended in fresh media in parallel showed proliferation like we have previously observed for each genotype (Figure 1B, Table 1), indicating the viability of cultures inoculated into conditioned media (data not shown). These results indicate that a factor or factors present in stationary conditioned medium prevents cell proliferation and that BzpN is necessary for the full activity of this factor.

## Discussion

AprA and CfaD are autocrine signals that inhibit proliferation, though little is known regarding their mechanism of action. We provide evidence here showing that the putative transcription factor BzpN is necessary for some of the responses to AprA and CfaD. *bzpN^−^* cells proliferate rapidly during exponential growth, as would be predicted for cells lacking downstream effectors of AprA or CfaD. However, in contrast to *aprA^−^* or *cfaD^−^* cells, *bzpN^−^* cells do not proliferate to a higher stationary density than wild-type cells. This suggests that AprA and CfaD may signal through a branched pathway, of which one branch functions to inhibit proliferation at lower densities and the other at higher densities. The BzpN-independent branch may not be active in low-density cells, which would explain the inability of high-density conditioned media to arrest the proliferation of low-density *bzpN^−^* cells (Figure 6). This model would predict the existence of mutants that proliferate like wild type cells at lower densities, but reach higher stationary densities than wild type. Mutants that show this phenotype have in fact been characterized [39].

Overexpression of BzpN-GFP in the wild type background resulted in a reduced maximum cell density as compared to wild type, whereas expression of BzpN-GFP in the *bzpN^−^* background resulted in a maximum cell density like wild type, suggesting that the native transcriptional or post-transcriptional regulation of *bzpN* strongly affects density-dependent inhibition of proliferation. This effect could be due to a density-dependent increase in BzpN expression, which would not occur in the *bzpN^−^/bzpN-GFP* strain, though further work is needed to evaluate this possibility. Overexpression of BzpN-GFP in *aprA^−^*, *cfaD^−^*, or *qkgA^−^* cells did not cause a significant reduction in cell density, indicating that these proteins are required for the inhibition of proliferation by BzpN. Additionally, these results strongly suggest that AprA, CfaD, and QkgA regulate BzpN activity by a mechanism other than transcriptional regulation, as BzpN-GFP is expressed in these cell lines but proliferation is not inhibited.

Intriguingly, we saw that, although rAprA and rCfaD inhibited the proliferation of wild-type cells, rAprA and rCfaD increased the proliferation of *bzpN^−^* cells, though only the increase in response to rCfaD was statistically significant. This result suggests the existence of a proliferation-promoting branch of the CfaD signal transduction pathway that serves to attenuate the effect of the pathway on proliferation and that BzpN is not a component of this branch. In *bzpN^−^* cells, the proliferation-promoting element of the pathway appears to still be active, thereby resulting in an increase of proliferation in response to AprA and CfaD.

We observed that BzpN-GFP fusion proteins showed a punctate intracellular localization at low cell density, and addition of conditioned media from high-density cells resulted in a nuclear localization of BzpN-GFP, indicating that an extracellular signal present at high cell density induces a localization of BzpN at the nucleus. However, this localization occurred in response to conditioned media from either *aprA^−^* or *cfaD^−^* cells, and did not occur in response to rAprA, rCfaD, or both proteins. These results suggest that an extracellular signal that is not AprA or CfaD is required for the nuclear translocation of BzpN. AprA and CfaD may activate BzpN by a mechanism independent of translocation, such as an activating phosphorylation that does not regulate subcellular localization, as is the case for the *Arabidopsis* bZIP transcription factor TRAB1 in response to the hormone abscisic acid [40]. Alternatively, a low basal level of nuclear BzpN may be permissive in regards to AprA and CfaD signaling, perhaps by allowing the expression of a component of the AprA/CfaD signal transduction pathway. In this model BzpN could be activated independently of AprA or CfaD, causing the high level of nuclear localization, but still could be necessary for AprA and CfaD function.

It is unclear why BzpN has been selected for during the evolution of *Dictyostelium*. *aprA^−^* and *cfaD^−^* cells show defects in spore generation [18], [19] and in the expansion of colonies on solid substrates [22], correlating the loss of these genes with a lack of fitness under some circumstances. However, *bzpN^−^* cells do not share these phenotypes, and thus BzpN likely functions to increase fitness in some other context. Intriguingly, expression of BzpN increases approximately 15-fold during the transition from growth to development [41], suggesting some role for BzpN during the developmental stage. BzpN may play a role in mitigating cellular stress, as cells lacking BzpN show aberrant proliferation under conditions of nitrosative or thermal stress (see Supporting Information S1, Figure S1, and Figure S2). Alternatively, BzpN may be functionally redundant with another protein affecting spore development or colony expansion, with the redundancy providing increased robustness.

Together, our data indicate that BzpN is necessary for the inhibition of proliferation by the autocrine signals AprA and CfaD, but that AprA and CfaD function in other processes that do not require BzpN. An important but unanswered question is how the putative transcription factor activity of BzpN might be altering gene expression to regulate cell proliferation. The tractability of *Dictyostelium* genetics may be useful in identifying potentially conserved genes that are upregulated or downregulated in response to BzpN activity that function to regulate proliferation, which may reveal novel approaches to reduce the proliferation of cancers.

## Supporting Information

Figure S1 **Transcription of** ***bzpN*** **is induced under stress conditions.** A semi-quantitative RT-PCR was performed with RNA samples collected under various stress conditions: vegetative growth, starvation in KK2 buffer, incubation in HL5 with 500 µM SNP and incubation in HL5 at 30°C for 24 hours. IG7 was used as a control.
(TIF)

Figure S2 **Proliferation of wild-type and** ***bzpN^−^*** **cells under stress conditions.** Exponentially growing cells were collected at 2×10^6^ cells/ml and diluted to 5×10^5^ cells/ml in HL5. (A) Cells were grown at 30°C to cause thermal stress. (B) 1 mM sodium nitroprusside (SNP) (Sigma) was added to the culture to introduce nitrosative stress and the cells were incubated at 22°C. Cells under all conditions were monitored by counting with hemocytometer over a week or until complete cell lysis was observed. Values are means ± SEM (n = 3). For proliferation at 30°C, differences in cell densities from days 2–7 are significant (p<0.001, t-test). For proliferation in the presence of SNP, differences in densities are significant at the 148-hour timepoint (p<0.05, one-tailed t-test).
(TIF)

Supporting Information S1 **Supplement: Other activities of** ***bzpN*** **.**
(DOC)

## References

[1] Kango-Singh M, Singh A (2009). Regulation of organ size: insights from the Drosophila Hippo signaling pathway.. Dev Dyn.

[2] Gomer RH (2001). Not being the wrong size.. Nat Rev Mol Cell Biol.

[3] Bullough WS, Hewett CL, Laurence EB (1964). The Epidermal Chalone; a Preliminary Attempt at Isolation.. Exp Cell Res.

[4] McPherron AC, Lawler AM, Lee SJ (1997). Regulation of skeletal muscle mass in mice by a new TGF-beta superfamily member.. Nature.

[5] Voaden MJ (1968). A chalone in the rabbit lens?. Exp Eye Res.

[6] Garcia-Giralt E, Lasalvia E, Florentin I, Mathe G (1970). Evidence for a lymphocytic chalone.. Rev Eur Etud Clin Biol.

[7] Houck JC, Weil RL, Sharma VK (1972). Evidence for a fibroblast chalone.. Nat New Biol.

[8] Guba M, Cernaianu G, Koehl G, Geissler EK, Jauch KW (2001). A primary tumor promotes dormancy of solitary tumor cells before inhibiting angiogenesis.. Cancer Res.

[9] Peeters CF, de Waal RM, Wobbes T, Ruers TJ (2008). Metastatic dormancy imposed by the primary tumor: does it exist in humans?. Ann Surg Oncol.

[10] Demicheli R (2001). Tumour dormancy: findings and hypotheses from clinical research on breast cancer.. Semin Cancer Biol.

[11] Brock DA, Gomer RH (1999). A cell-counting factor regulating structure size in Dictyostelium.. Genes Dev.

[12] Jain R, Yuen IS, Taphouse CR, Gomer RH (1992). A density-sensing factor controls development in Dictyostelium.. Genes Dev.

[13] Gomer RH, Yuen IS, Firtel RA (1991). A secreted 80×10(3) Mr protein mediates sensing of cell density and the onset of development in Dictyostelium.. Development.

[14] Manahan CL, Iglesias PA, Long Y, Devreotes PN (2004). Chemoattractant signaling in dictyostelium discoideum.. Annu Rev Cell Dev Biol.

[15] Kessin RH (2001). Dictyostelium : evolution, cell biology, and the development of multicellularity.

[16] Aubry L, Firtel R (1999). Integration of signaling networks that regulate Dictyostelium differentiation.. Annu Rev Cell Dev Biol.

[17] Bonner JT (2003). Evolution of development in the cellular slime molds.. Evolution & Development.

[18] Brock DA, Gomer RH (2005). A secreted factor represses cell proliferation in Dictyostelium.. Development.

[19] Bakthavatsalam D, Brock DA, Nikravan NN, Houston KD, Hatton RD (2008). The secreted Dictyostelium protein CfaD is a chalone.. J Cell Sci.

[20] Choe JM, Bakthavatsalam D, Phillips JE, Gomer RH (2009). Dictyostelium cells bind a secreted autocrine factor that represses cell proliferation.. BMC Biochem.

[21] Bakthavatsalam D, Choe JM, Hanson NE, Gomer RH (2009). A Dictyostelium chalone uses G proteins to regulate proliferation.. BMC Biol.

[22] Phillips JE, Gomer RH (2010). The ROCO kinase QkgA is necessary for proliferation inhibition by autocrine signals in Dictyostelium discoideum.. Eukaryot Cell.

[23] Kerr LD, Inoue J, Verma IM (1992). Signal transduction: the nuclear target.. Curr Opin Cell Biol.

[24] Jakoby M, Weisshaar B, Droge-Laser W, Vicente-Carbajosa J, Tiedemann J (2002). bZIP transcription factors in Arabidopsis.. Trends in Plant Science.

[25] Landschulz WH, Johnson PF, McKnight SL (1988). The leucine zipper: a hypothetical structure common to a new class of DNA binding proteins.. Science.

[26] Busch SJ, Sassone-Corsi P (1990). Dimers, leucine zippers and DNA-binding domains.. Trends Genet.

[27] Huang E, Blagg SL, Keller T, Katoh M, Shaulsky G (2006). bZIP transcription factor interactions regulate DIF responses in Dictyostelium.. Development.

[28] Thompson CR, Fu Q, Buhay C, Kay RR, Shaulsky G (2004). A bZIP/bRLZ transcription factor required for DIF signaling in Dictyostelium.. Development.

[29] Zhukovskaya NV, Fukuzawa M, Yamada Y, Araki T, Williams JG (2006). The Dictyostelium bZIP transcription factor DimB regulates prestalk-specific gene expression.. Development.

[30] Faix J, Kreppel L, Shaulsky G, Schleicher M, Kimmel AR (2004). A rapid and efficient method to generate multiple gene disruptions in Dictyostelium discoideum using a single selectable marker and the Cre-loxP system.. Nucleic Acids Res.

[31] Ahern KG, Howard PK, Firtel RA (1988). Identification of regions essential for extrachromosomal replication and maintenance of an endogenous plasmid in Dictyostelium.. Nucleic Acids Res.

[32] Veltman DM, Akar G, Bosgraaf L, Van Haastert PJ (2009). A new set of small, extrachromosomal expression vectors for Dictyostelium discoideum.. Plasmid.

[33] Manstein DJ, Schuster HP, Morandini P, Hunt DM (1995). Cloning vectors for the production of proteins in Dictyostelium discoideum.. Gene.

[34] Bokko PB, Francione L, Bandala-Sanchez E, Ahmed AU, Annesley SJ (2007). Diverse cytopathologies in mitochondrial disease are caused by AMP-activated protein kinase signaling.. Mol Biol Cell.

[35] Sykiotis GP, Bohmann D (2010). Stress-activated cap'n'collar transcription factors in aging and human disease.. Sci Signal.

[36] Su TT, O'Farrell PH (1998). Size control: cell proliferation does not equal growth.. Curr Biol.

[37] Chen G, Shaulsky G, Kuspa A (2004). Tissue-specific G1-phase cell-cycle arrest prior to terminal differentiation in Dictyostelium.. Development.

[38] Yarger J, Stults K, Soll DR (1974). Observations on the growth of Dictyostelium discoideum in axenic medium: evidence for an extracellular growth inhibitor synthesized by stationary phase cells.. J Cell Sci.

[39] Souza GM, Lu S, Kuspa A (1998). YakA, a protein kinase required for the transition from growth to development in Dictyostelium.. Development.

[40] Kagaya Y, Hobo T, Murata M, Ban A, Hattori T (2002). Abscisic acid-induced transcription is mediated by phosphorylation of an abscisic acid response element binding factor, TRAB1.. Plant Cell.

[41] Rot G, Parikh A, Curk T, Kuspa A, Shaulsky G (2009). dictyExpress: a Dictyostelium discoideum gene expression database with an explorative data analysis web-based interface.. BMC Bioinformatics.

