# Supplementary figures and images for: The Putative bZIP Transcripton Factor BzpN Slows Proliferation and Functions in the Regulation of Cell Density by Autocrine Signals in Dictyostelium
Source: PLoS One. 2011 Jul 7;6(7):e21765. doi: 10.1371/journal.pone.0021765 (PMC3131300; doi:10.1371/journal.pone.0021765)

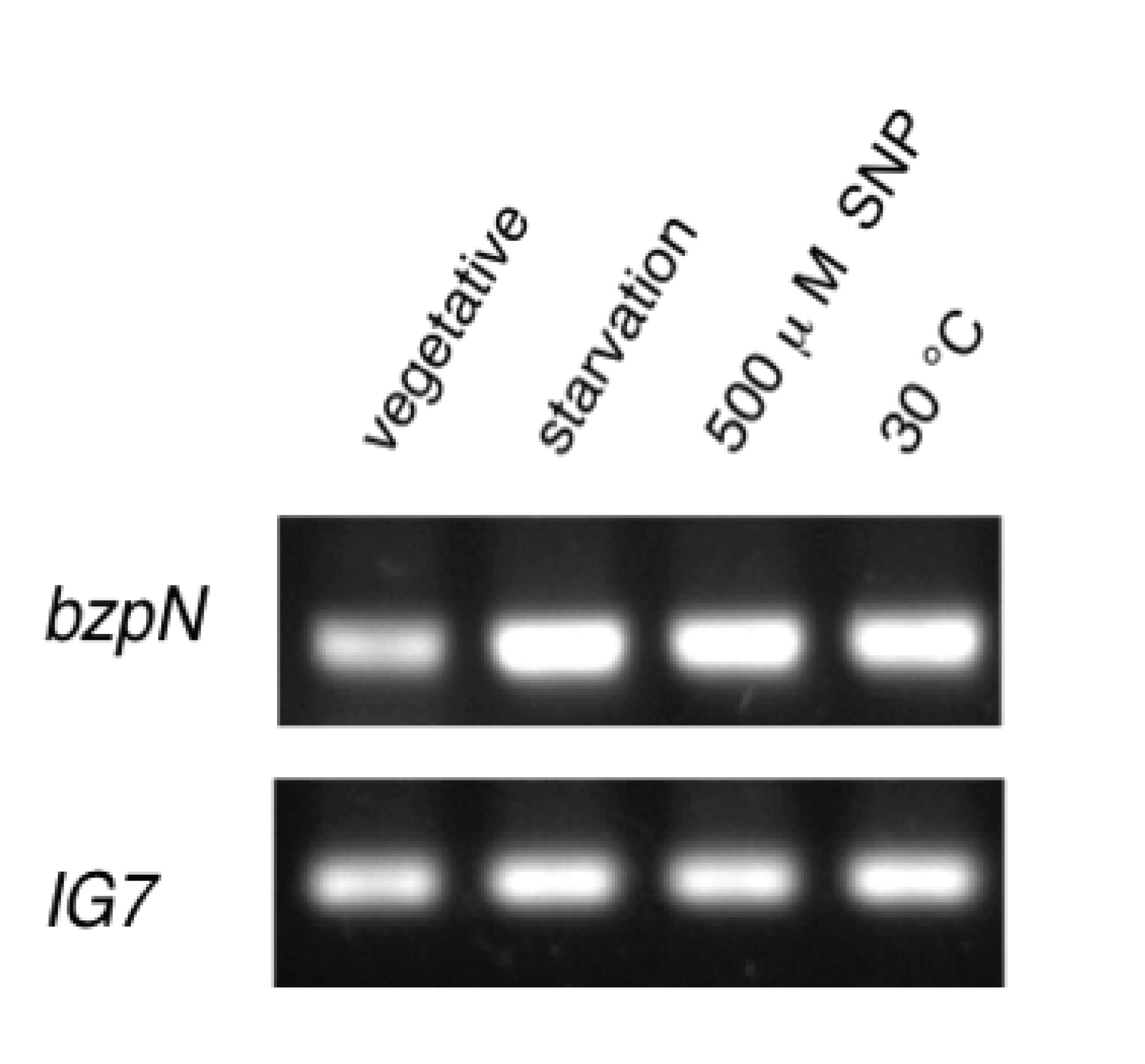

Supplement: Figure S1 — Transcription of bzpN is induced under stress conditions. A semi-quantitative RT-PCR was performed with RNA samples collected under various stress conditions: vegetative growth, starvation in KK2 buffer, incubation in HL5 with 500 µM SNP and incubation in HL5 at 30°C for 24 hours. IG7 was used as a control. (TIF) [file pone.0021765.s001.tif]

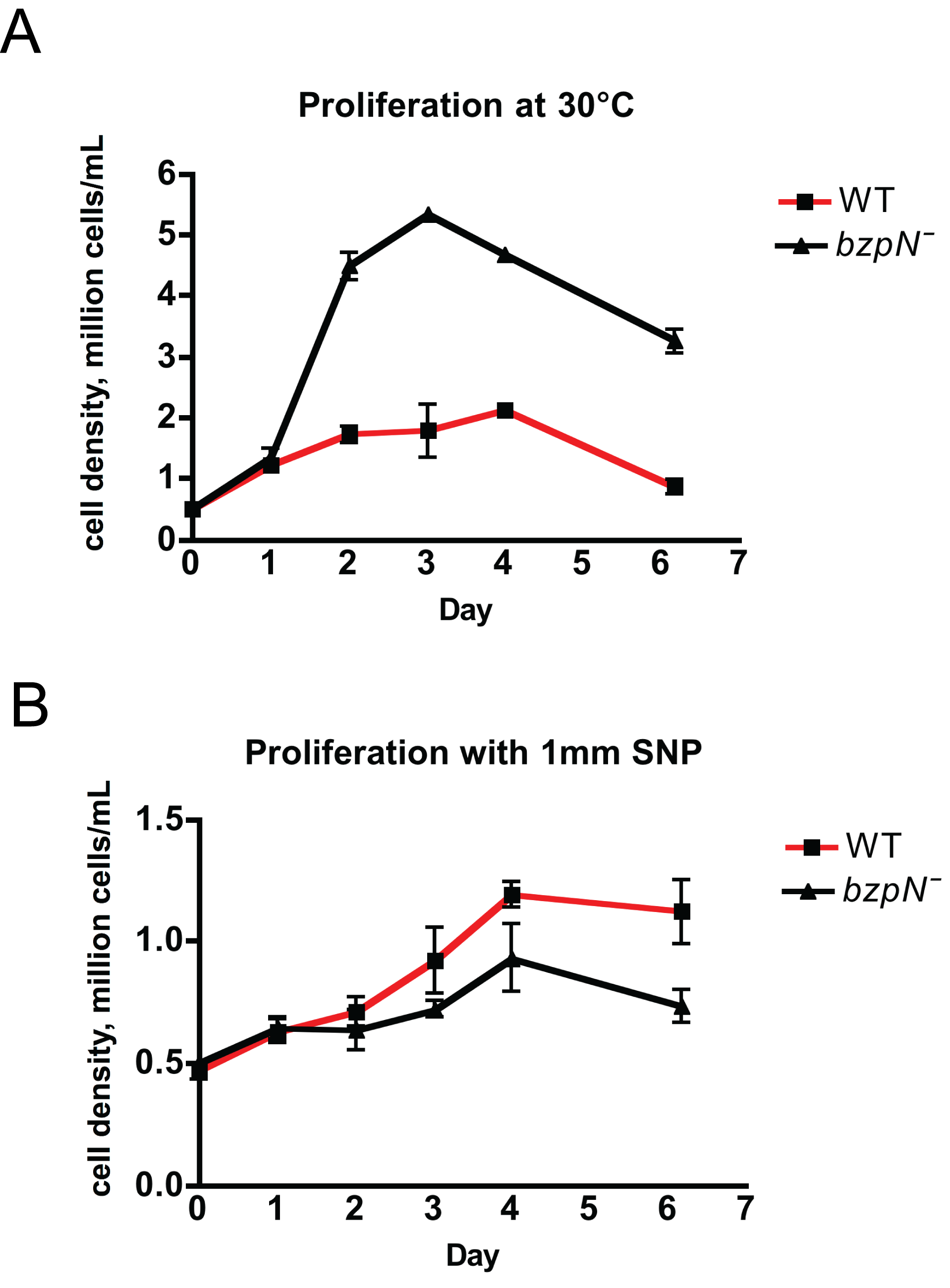

Supplement: Figure S2 — Proliferation of wild-type and bzpN− cells under stress conditions. Exponentially growing cells were collected at 2×10^6^ cells/ml and diluted to 5×10^5^ cells/ml in HL5. (A) Cells were grown at 30°C to cause thermal stress. (B) 1 mM sodium nitroprusside (SNP) (Sigma) was added to the culture to introduce nitrosative stress and the cells were incubated at 22°C. Cells under all conditions were monitored by counting with hemocytometer over a week or until complete cell lysis was observed. Values are means ± SEM (n = 3). For proliferation at 30°C, differences in cell densities from days 2–7 are significant (p<0.001, t-test). For proliferation in the presence of SNP, differences in densities are significant at the 148-hour timepoint (p<0.05, one-tailed t-test). (TIF) [file pone.0021765.s002.tif]
