# Supplementary material for: The Putative bZIP Transcripton Factor BzpN Slows Proliferation and Functions in the Regulation of Cell Density by Autocrine Signals in Dictyostelium
Source: PLoS One. 2011 Jul 7;6(7):e21765. doi: 10.1371/journal.pone.0021765 (PMC3131300; doi:10.1371/journal.pone.0021765)
Supplement: Supporting Information S1 — Supplement: Other activities of bzpN . (DOC) [file pone.0021765.s003.doc]

**Supplement: Other activities of *bzpN***

Based on the RNA-seq transcriptome of *D. discoideum*, *bzpN* mRNA is present in bacteria-fed vegetative cells in small amounts and its level is increased about 20-fold during development, peaking at 20 hours with a 4-fold enrichment in prestalk cells (Parikh et al., 2010 and <http://www.ailab.si/dictyexpress/>).

The predicted BzpN protein sequence is similar to the sequence of the yeast GCN4, which is a central regulator of stress responses. We found that *bzpN* mRNA levels are increased upon starvation, nitrosative stress caused by sodium nitroprusside (500 µM SNP, Sigma) and heat stress (30°C) (Supplement Figure S1). *bzpN* also appears to have a direct role in the cells’ ability to handle stress. Comparing the cell density of growing cultures that were incubated at 30°C (the optimal growth temperature for *D. discoideum* is 22°C) or in the presence of 1mM SNP, the *bzpN¯* mutants were different from the wild type. At 30°C the mutant cells divided with a much shorter doubling time than the wild type. They reached a higher cell density after 72 hours but then started to die at a rapid rate (Supplement Figure S2A). Both wild type and mutant cells eventually died after prolonged incubation at 30°C (data not shown). Under nitrosative stress, the mutant cells divided more slowly than the wild type and reached a lower saturation density (Supplement Figure S2B).

**References**

Parikh, A., Miranda, E.R., Katoh-Kurasawa, M., Fuller, D., Rot, G., Zagar, L., Curk, T., Sucgang, R., Chen, R., Zupan, B.*, et al.* (2010). Conserved developmental transcriptomes in evolutionarily divergent species. Genome Biol *11*, R35.
